# Human papillomavirus type 18 E5 oncogene supports cell cycle progression and impairs epithelial differentiation by modulating growth factor receptor signalling during the virus life cycle

**DOI:** 10.18632/oncotarget.21658

**Published:** 2017-10-06

**Authors:** Christopher W. Wasson, Ethan L. Morgan, Marietta Müller, Rebecca L. Ross, Margaret Hartley, Sally Roberts, Andrew Macdonald

**Affiliations:** ^1^ School of Molecular and Cellular Biology, Astbury Centre for Structural and Molecular Biology, Faculty of Biological Sciences, University of Leeds, Leeds, UK; ^2^ Institute of Cancer and Genomic Sciences, College of Medical and Dental Sciences, University of Birmingham, Birmingham, UK

**Keywords:** HPV, E5, proliferation, EGFR, differentiation

## Abstract

Deregulation of proliferation and differentiation-dependent signalling pathways is a hallmark of human papillomavirus (HPV) infection. Although the manipulation of these pathways by E6 and E7 has been extensively studied, controversies surround the role of the E5 oncoprotein during a productive virus life cycle. By integrating primary keratinocytes harbouring wild type or E5 knockout HPV18 genomes with pharmacological and gain/loss of function models, this study aimed to provide molecular information about the role of E5 in epithelial proliferation and differentiation. We show that E5 contributes to cell cycle progression and unscheduled host DNA synthesis in differentiating keratinocytes. E5 function correlates with increased EGFR activation in differentiating cells and blockade of this pathway impairs differentiation-dependent cell cycle progression of HPV18 containing cells. Our findings provide a functional requirement of enhanced EGFR signalling for suprabasal cellular DNA synthesis during the virus life cycle. They also reveal an unrecognised contribution of E5 towards the impaired keratinocyte differentiation observed during a productive HPV infection. E5 suppresses a signalling axis consisting of the keratinocyte growth factor receptor (KGFR) pathway. Inhibition of this pathway compensates for the loss of E5 in knockout cells and re-instates the delay in differentiation. The negative regulation of KGFR involves suppression by the EGFR pathway. Thus our data reveal an unappreciated role for E5-mediated EGFR signalling in orchestrating the balance between proliferation and differentiation in suprabasal cells.

## INTRODUCTION

Human papillomaviruses (HPV) infect the squamous epithelial cells at a number of body sites [1, 2]. A small number of HPV types are carcinogenic and recognized as the agent for most anogenital cancers and a subset of cancers of the oropharyngeal tract, with HPV16 and HPV18 being responsible for the largest incidence of cervical cancer [3, 4].

Keratinocyte homeostasis is controlled by the complex interplay of signalling pathways functioning in an integrated fashion. In the basal compartment, epidermal growth factor receptor (EGFR) signalling is a major determinant of keratinocyte proliferation by promoting cell cycle progression [5]. Tissues lacking key components of the EGFR signalling pathway including ERK/MAPK are hypoproliferative and display G2/M arrest [5]. In addition to increasing proliferation, EGFR signalling suppresses keratinocyte differentiation [6]. This is required to maintain homeostasis between self-renewing and committed keratinocytes in the basal proliferative compartment of the epidermis, whereas in the upper layers this pathway is down regulated. Once a keratinocyte enters the suprabasal layer, it induces expression of proteins involved in the sequential programme of terminal differentiation [7]. Whilst there is not a complete understanding of the pathways that orchestrate differentiation, a number of critical regulators of this process are recognized including the keratinocyte growth factor receptor (KGFR/FGFR2IIIb). This is expressed in suprabasal layers and induces gene expression changes associated with terminal differentiation. KGFR over-expression in basal cells induces premature expression of spinous layer associated markers, whilst ablation, by genetic or chemical means, results in a hyperproliferative epidermis [8, 9].

The HPV life cycle is linked to differentiation of the epithelial tissues they infect. Following infection of keratinocytes in the epithelial basal layer, HPV genomes are established as episomes. After a mitotic event, infected basal cells migrate towards the suprabasal regions and begin to differentiate. In contrast to uninfected keratinocytes, when the infected basal cells detach from the basement membrane they fail to undergo the early stages of differentiation and re-enter the cell cycle in the suprabasal layers to enable the virus episomes to be amplified to a high copy number [1]. In the upper layers of the epithelium, infected cells exit the cell cycle and re-enter the differentiation process, enabling transit to the late stage of infection, where the late promoter is activated to drive expression of the structural proteins in preparation for packaging of the viral genome into newly synthesized capsids.

High-risk HPV genomes encode three early genes (E5, E6 and E7), classed as oncogenes that manipulate the host cell environment by influencing cell proliferation, differentiation and survival. E6 and E7 are the major drivers of keratinocyte proliferation and their expression is retained in cancers. They are required for immortalization of keratinocytes and their best understood functions are to inactivate p53 and pRb tumour suppressor proteins [10, 11]. In contrast, E5 remains one of the least understood of the early proteins [12]. E5 is a membrane-integrated protein [12, 13] presumed to localize to the endoplasmic reticulum and Golgi apparatus [14]. Efforts to understand the contribution of E5 to HPV pathogenesis have shown that it induces anchorage-independent growth in murine fibroblasts and growth in low serum [15, 16], whilst demonstrating weak transforming activity in primary human keratinocytes [17]. Several studies have shown that E5 potentiates EGFR signalling, and modulation of this pathway is implicated in its transforming ability [12, 18]. Transgenic mouse models expressing HPV16 E5 in the epithelium display hyperplasia, resulting in spontaneous tumour formation [19]. These mice also display increased dysplastic disease in the cervical epithelium [20]. Importantly, the epithelial hyperplasia induced by E5 is attenuated when the EGFR pathway is inhibited by expression of a dominant negative EGFR [19]. In cervical cancers HPV genomes are regularly integrated into host DNA, and this occurs such that the *E5* gene is often lost. This suggests that E5 plays a critical role in the genesis of cervical cancer but less of a role in its progression or persistence.

Studies of E5 function in high-risk HPV16 [21] and HPV31 [22] life cycle models show that E5 function is likely not required by the virus in undifferentiated cells, but does play a role during the productive stages of infection in the differentiated epithelium. They highlight a need for E5 in regulating host cell cycle progression and aiding virus genome amplification. Despite these advances, the mechanisms by which E5 regulates these processes are unknown [23, 24]. Interestingly, neither study identified any differences in suprabasal differentiation in the absence of E5. These findings are disputed by more recent *in vivo* studies, highlighting a role for E5 in the deregulation of differentiation in the epithelia of the HPV16 transgenic mouse [19]. Subtle differences are also observed in the requirement for E5 between the two HPV types tested. These might relate to differences in experimental design, or the use of immortalized keratinocytes to study HPV16 versus primary keratinocytes to test HPV31. Alternatively, they may relate to genuine type specific differences in the role of E5. Finally, no apparent role for EGFR signalling was identified in either model, which is distinct from evidence supporting manipulation of this pathway in cells expressing E5, or the requirement for EGFR in E5-mediated transformation shown in the transgenic mouse model.

Given these disparities and the possibility of HPV type specific differences in E5 function, we examined the role of E5 in the HPV18 life cycle utilizing a primary human keratinocyte model system [25–27]. We confirm that loss of E5 function impacts on the productive stages of the virus life cycle and impairs maintenance of the cell cycle upon keratinocyte differentiation. We provide new evidence highlighting a role for E5 in impairing keratinocyte differentiation. At the biochemical level, E5 suppresses the KGFR pathway, whilst enhancing proliferative signalling. Use of small molecule inhibitors and expression of mutant signalling proteins affirms that keratinocyte differentiation requires an integrated signalling response with significant cross talk between pathways. In particular, attenuation of EGFR signalling impacted on all pathways studied. These data indicate that E5 subverts EGFR signalling as a unifying mechanism to alter proliferation and differentiation pathways in keratinocytes.

## RESULTS

### Loss of E5 expression does not alter HPV18 genome establishment in undifferentiated primary human keratinocytes

**Figure 1:**
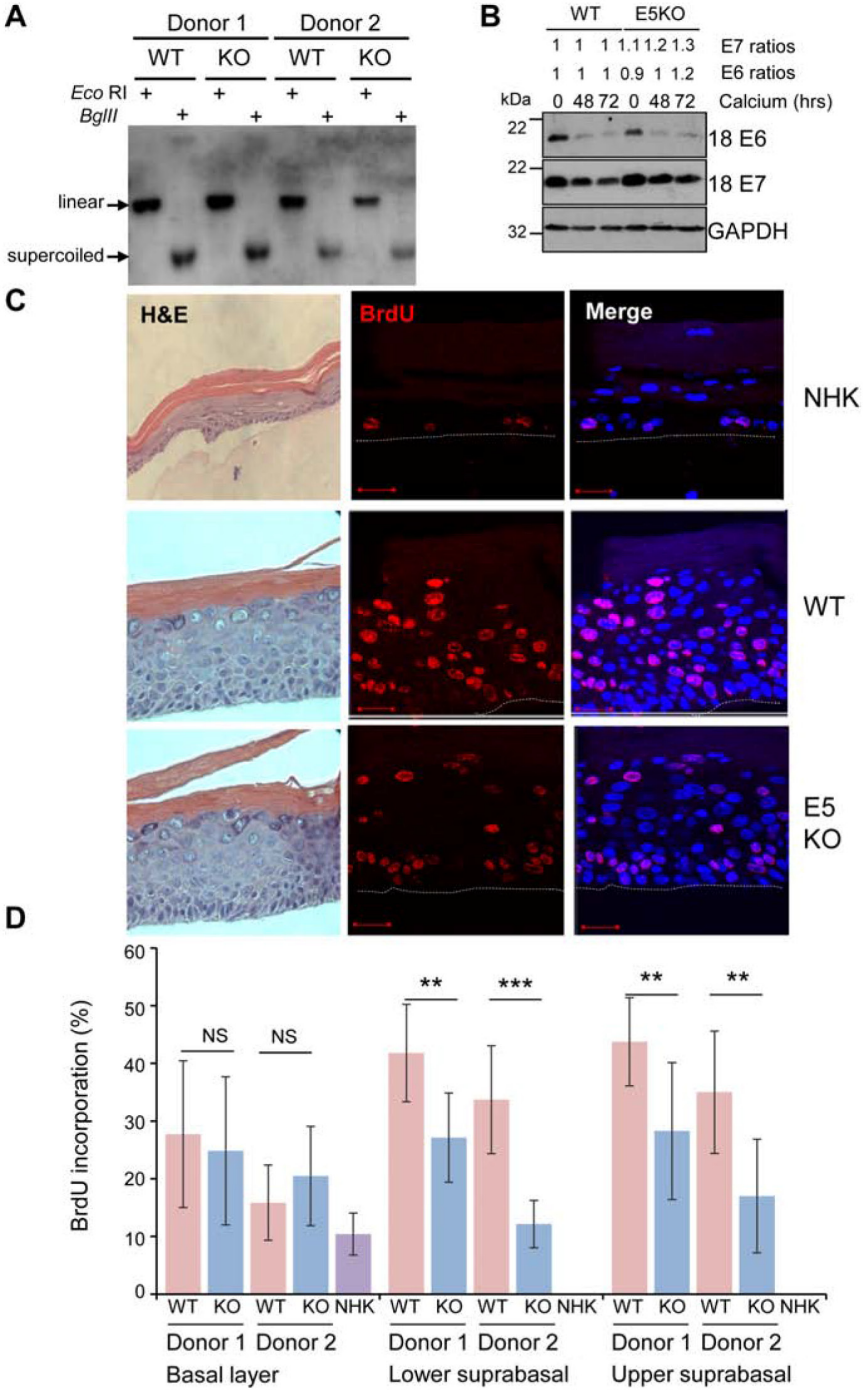
HPV18 E5 contributes towards unscheduled host cell DNA synthesis upon keratinocyte differentiation **(A)** Southern analysis of equal amounts of total DNA extracted from NHK transfected with wild-type (WT) or mutant (E5KO) genomes in two different donors. DNA was digested with *Dpn*I (digests input DNA) together with *Bgl*II which does not digest HPV18 DNA or *Eco*RI which linearizes HPV18 genomes. **(B)** Detection of E6 and E7 proteins in equal amounts of Triton-X100 detergent soluble protein lysates prepared from cells differentiated in high-calcium monolayer cultures. Levels of GAPDH were used as a loading control. Densitometry analysis of protein bands was performed using ImageJ software. **(C)** Organotypic rafts were incubated with BrdU to identify nuclei positive for cellular DNA synthesis. BrdU positive cells were visualised with an anti-BrdU antibody (red) and nuclei visualised with DAPI (blue). White dotted lines indicate the basal cell layer. Red scale bar represents 20 μm. Organotypic raft cultures were also stained with haematoxylin and eosin to observe the gross morphology of the epithelium. **(D)** Graphs showing the percentage of BrdU positive nuclei in basal and lower suprabasal and upper suprabasal layers. The data, shown as a mean with standard deviation, were derived from 15 fields of view of each raft and from 3 independent experiments from two donors cell lines. Significance as determined by Student’s t-test is shown as ^**^ = p<0.01, ^***^ = p<0.001.

To study the role of E5 in the HPV18 life cycle, we generated a mutant HPV18 genome in which the E5 open reading frame was disrupted by the introduction of a translation termination codon. This mutant, HPV18 E5KO, contains a single nucleotide change at position 3940, inserting a translational stop codon at position 2 in the E5 sequence. The mutation would not be expected to interfere with any of the splice sites recently identified in the HPV18 genome [23, 28]. Wild type (WT) and E5KO (KO) HPV18 genomes were transfected into low passage neonate normal human keratinocytes (NHK) obtained from two individual foreskin donors. Southern blotting of total genomic DNA isolated from undifferentiated monolayer cultures showed that the WT and E5KO viral genomes were established as extra-chromosomal episomes (Figure 1A). Whilst minor differences exist in absolute genome copy number per cell in each donor, no significant differences were observed between the WT (donor 1: 172 and donor 2: 208) and E5KO (donor 1: 168 and donor 2: 194) genome-containing cells. After serial passaging of the cell lines (average population doubling times: WT, 34 hours; E5KO, 31 hours) the HPV18 episomes were maintained in the absence of E5 protein (data not shown). Whilst no antibody exists to detect E5, to ensure that the mutagenesis strategy did not adversely affect E6 and/or E7 expression, the levels of the two oncoproteins were assessed in lysates from cells cultured in high calcium media for over 72 hours (Figure 1B). Levels of both oncoproteins were highest in undifferentiated WT and E5KO cells and upon differentiation the levels of both proteins declined. Importantly, there was little variation of either oncoprotein between WT and E5KO cell lines under study.

### E5 supports cell cycle re-entry of post-mitotic suprabasal keratinocytes

Given a lack of requirement for E5 function in the early stages of the HPV18 life cycle we investigated its role during the differentiation-dependent stage of the infectious cycle. At this stage, the virus is required to re-establish cell cycle progression in the post-mitotic suprabasal cells to stimulate unscheduled host cell DNA synthesis to provide an environment conducive to virus genome amplification. To investigate the effect of E5 function on host DNA replication, HPV18 genome-containing lines were stratified in organotypic raft cultures for 13 days and the thymidine analogue bromodeoxyuridine (BrdU) was incorporated over the final 16 hours before fixation. The percentages of BrdU-positive cells present in the raft cultures were then quantified. As expected, BrdU-positive cells were confined to the basal layer in NHK-derived rafts, whereas BrdU-positive nuclei were detected in both the basal and suprabasal compartments of WT and E5KO genome containing cells (Figure 1C). Quantification of the number of BrdU positive nuclei revealed no significant differences in BrdU staining in the basal layers in E5KO cells compared to WT (Figure 1C and 1D). In contrast, a statistically significant decrease in BrdU staining was observed in the suprabasal layers in the E5KO rafts, which was consistent between donors (Figure 1C and 1D). Despite the decrease in DNA synthesis observed in the E5KO rafts, this did not equate to gross morphological changes in the rafts as observed in haematoxylin and eosin stained sections (Figure 1C).

**Figure 2:**
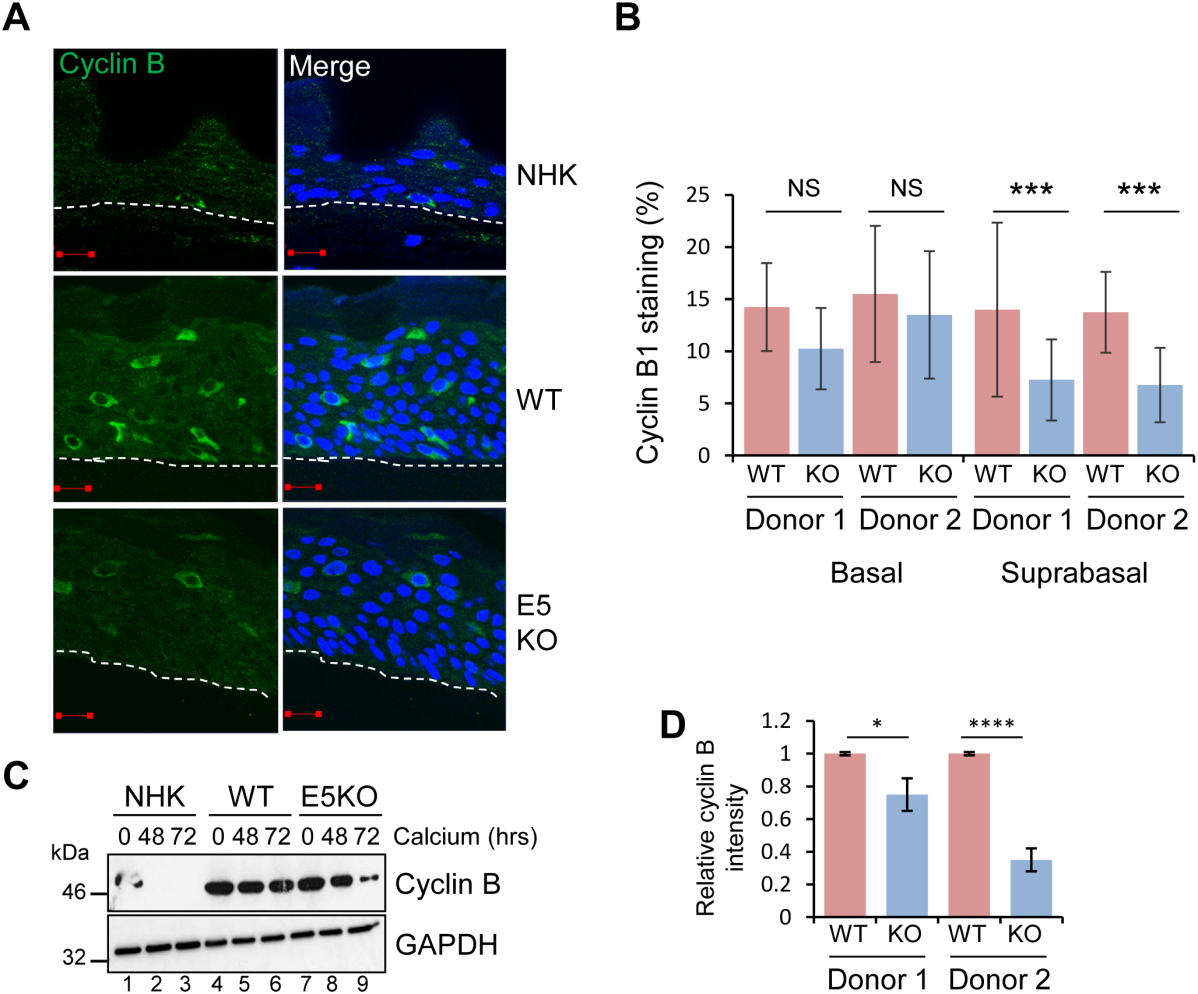
HPV18 E5 deregulates cell cycle progression in differentiated cells **(A)** Organotypic rafts stained for cyclin B1 (green) and nuclei stained with DAPI (blue), white dotted lines indicate the basal cell layer. Red scale bar represents 20 μm. **(B)** Graphs showing the percentage of cyclin B1 positive nuclei in basal and suprabasal layers. The data, shown as a mean with standard deviation, were derived from 15 fields of view of each raft and from 3 independent experiments from two donor cell lines. Significance as determined by Student’s t-test is shown as ^***^ = p<0.001. **(C)** Lysates of NHK, WT and E5KO cells subjected to high calcium differentiation were analysed for cyclin B1 expression by immunoblotting. GAPDH served as a loading control. **(D)** Graph showing densitometry analysis of cyclin B1 expression at 72 hours for two donors. The data is shown as a mean and standard deviation from three independent experiments. Significance as determined by Student’s t-test is shown as ^*^ = p<0.05 and ^****^ = p<0.0001.

Recent observations suggest that HPV DNA amplification occurs in suprabasal cells that have entered a protracted G2 phase of the cell cycle [29]. Cells in G2 are identified by cytoplasmic cyclin B1 expression; the cyclin-cdk complex is retained in the cytoplasm in an inactive form and must first be dephosphorylated prior to the nuclear translocation observed during mitosis. To determine if E5 function was necessary for progression of the viral genome-containing cells through to G2, cyclin B1 expression was assessed. Cytoplasmic cyclin B1 was restricted to the basal layer of NHK raft cultures (Figure 2A). In the WT and E5KO cultures, cytoplasmic cyclin B1 was detected in the basal and suprabasal layers (Figure 2A and 2B). Whilst levels of cyclin B1 positive stained cells in the basal layer did not differ, we observed a significant loss of cyclin B1 staining in the suprabasal layers of the E5KO rafts (Figure 2A and 2B).

These data were confirmed in cells differentiated in high calcium media for 72 hours. Cyclin B1 levels declined rapidly in differentiated NHK (Figure 2C: lanes 1-3). In accordance with observations from raft cultures cyclin B1 expression persisted in differentiating keratinocytes harbouring HPV18 WT and E5KO cells (Figure 2C lanes 4-6, 7-9). However, at 72 hours the level of cyclin B1 in the E5KO cells was significantly reduced compared to WT (Figure 2C; compare lanes 6 and 9) (Quantification in Figure 2D). Taken together, these data suggest that E5 contributes to the maintenance of cell cycle activity in suprabasal cells.

### E5 is not necessary for HPV18 genome amplification or late viral protein expression in differentiating keratinocytes

**Figure 3:**
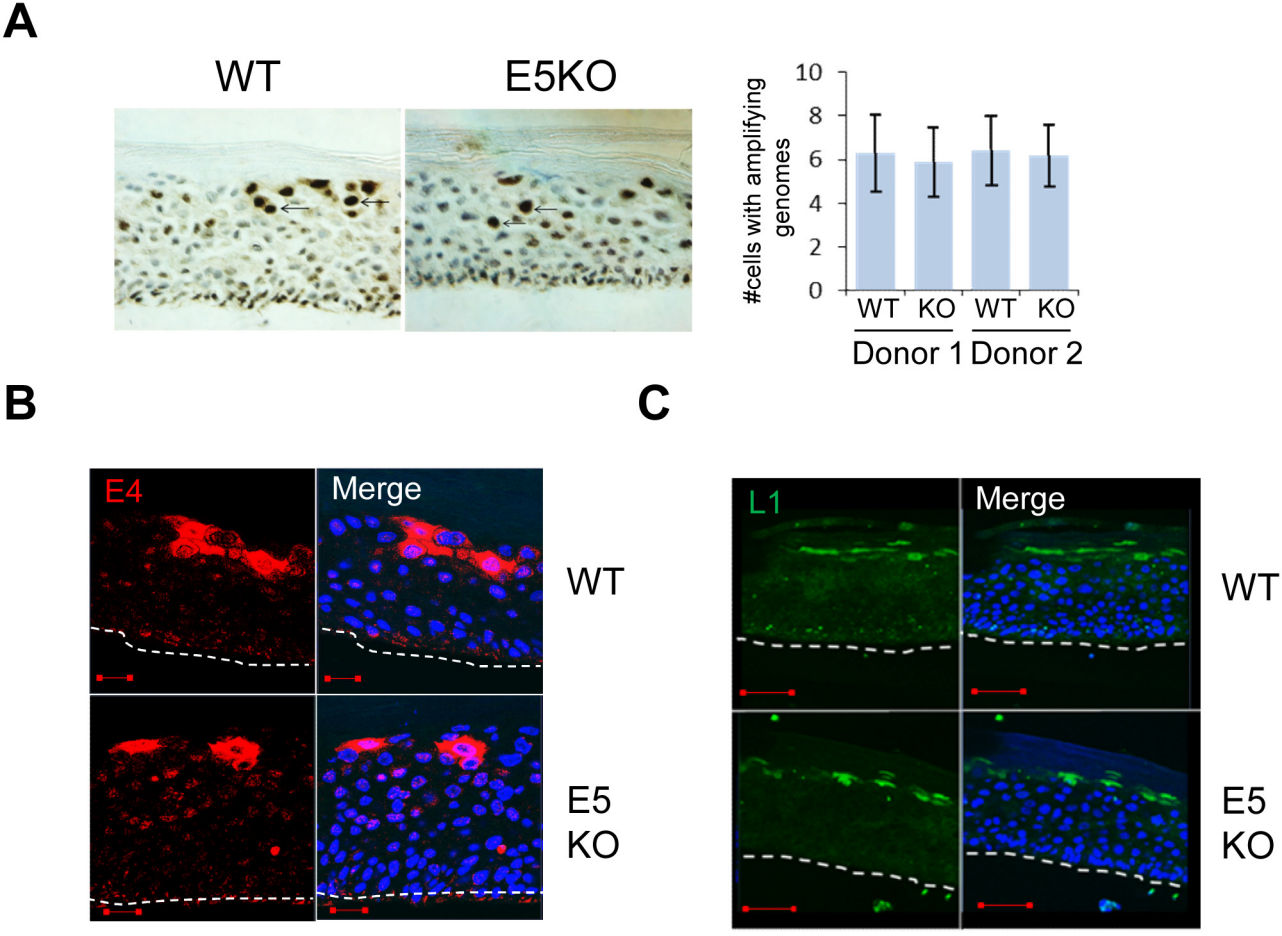
HPV18 E5 function is not necessary for genome amplification and late protein expression in differentiating keratinocytes **(A)** Organotypic raft cultures were probed with a biotin-conjugated HPV DNA specific for the high-risk HPV types to visualise genome amplification. Arrows indicate examples of CISH positive nuclei. Graph represents the mean (± standard deviation) of CISH positive nuclei per field of view (15 fields of view of each raft) for two separate donors. **(B** and **C)** Organotypic rafts stained for E4 (red) and L1 (green), nuclei were visualised with DAPI (blue). White dotted lines indicate the basal cell layer. Red scale bar represents 20 μm. Images are representative of staining from two donors and three independent experiments.

Given that our data indicated a decrease in the ability of cells containing the E5KO genomes to retain proliferative potential once exposed to differentiation stimuli, we examined viral genome amplification by chromogenic *in situ* hybridisation (CISH) using a HPV specific probe on raft sections. As shown in Figure 3A, intensely stained nuclei, corresponding to cells undergoing viral DNA amplification, were found in cells of the upper suprabasal layers of the rafts carrying WT and E5KO genomes. Quantification of the data did not show a significant difference in the number of cells undergoing viral genome amplification in the absence of E5 (Figure 3A). These results indicate that whilst loss of E5 impacts on host suprabasal DNA synthesis, it does not make a significant contribution towards virus genome amplification.

Expression of the virus capsid proteins and the E4 protein occurs in the more differentiated cells of the epithelium. To determine if loss of E5 affected these hallmarks of the productive life cycle, rafts were stained with antibodies that recognise E4, an HPV protein that is up-regulated at the onset of viral genome amplification (Figure 3B), and the major capsid protein L1 (Figure 3C). In rafts of cells containing the WT genomes staining of E4 and L1 was observed in the upper suprabasal layers. No significant differences in the staining patterns were observed between WT and E5KO cells. Taken together, these data indicate that HPV18 E5 is not required for the expression of late viral proteins and concurs with previous findings from the HPV16 life cycle model lacking E5 expression [21].

### HPV18 E5 enhances EGFR expression in primary human keratinocytes

**Figure 4:**
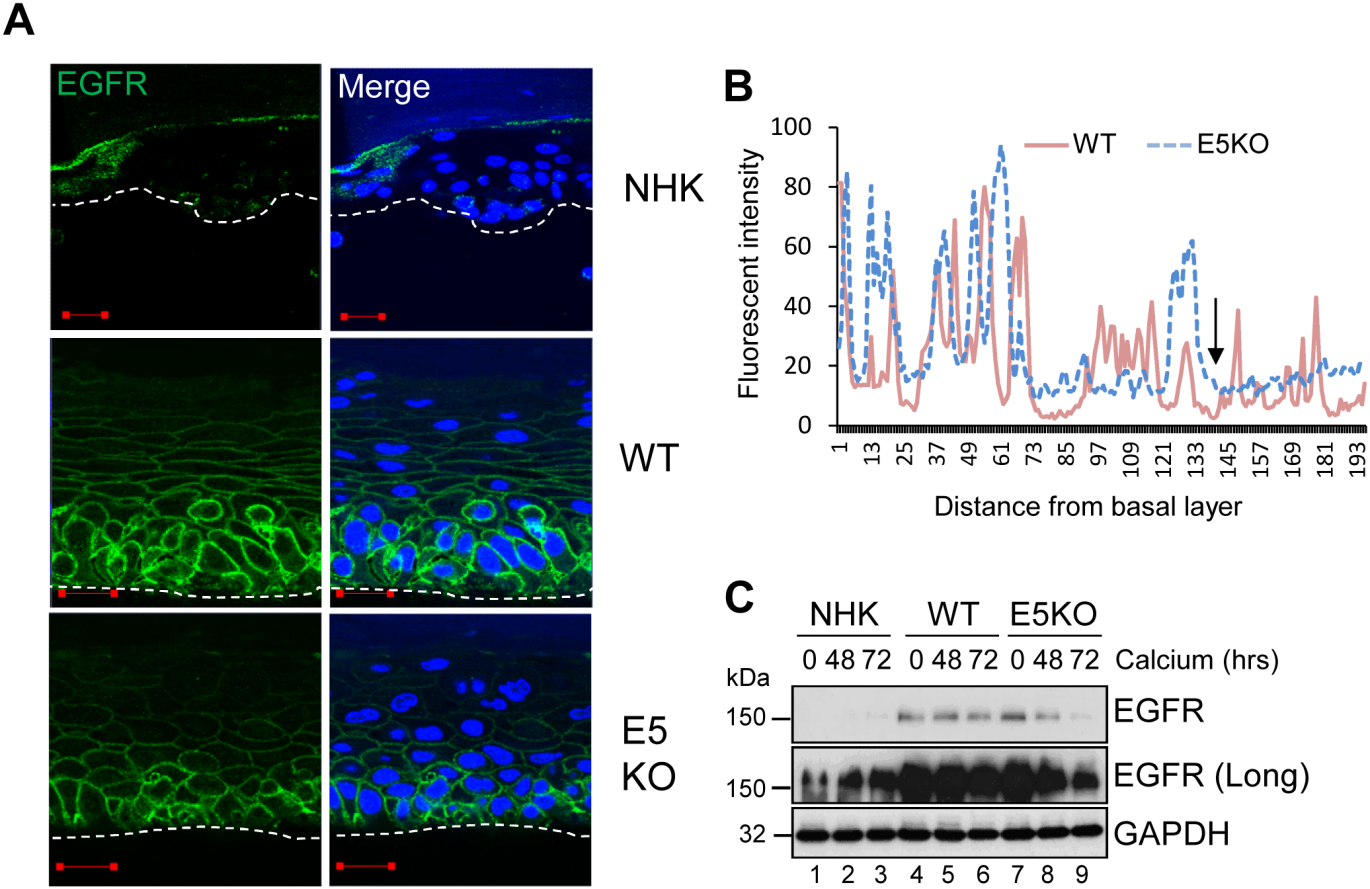
HPV18 E5 maintains EGFR expression in differentiating keratinocytes **(A)** Organotypic rafts stained for EGFR (green), with nuclei stained with DAPI (blue). White dotted lines indicate the basal cell layer. Red scale bar represents 20 μm. Images are representative of staining from two donors. **(B)** Histogram analysis of the staining intensity from WT and E5KO rafts. Data was derived from 5 fields of view from three independent experiments and two donors. **(C)** Lysates of keratinocytes differentiated in high calcium media analysed for total EGFR expression by immunoblotting. GAPDH served as a loading control. Western blots are representative of three independent experiments in two donor lines.

EGFR signalling is a major driver of proliferation in the basal compartment of the epithelium [6]. It regulates the activity of downstream components such as ERK/MAPK, which are essential for G2/M progression in primary keratinocytes [5]. Expression of isolated E5 has been shown to augment EGFR expression and autophosphorylation, and to enhance mitogenic signalling [30]. To determine if the effects of E5 on the cell cycle progression of post mitotic keratinocytes was linked to an effect on EGFR signalling during the differentiation dependent life cycle, NHK and HPV18 genome-containing keratinocytes were stratified in raft culture and levels of total EGFR detected by immunofluorescence staining. EGFR expression was restricted to the basal cells in normal human keratinocytes (Figure 4A). In contrast, WT genome containing rafts exhibited a marked increase in plasma membrane localised EGFR expression in the basal and suprabasal layers (Figure 4A). E5KO rafts also displayed increased plasma membrane localised EGFR expression in the basal compartment, however, suprabasal EGFR expression was reduced compared to WT rafts (Figure 4A). WT and E5KO rafts were digitally scanned from basal layer to the top and analysed by cross-sectional imaging analysis (Figure 4B). No obvious difference was observed in the EGFR expression profile in the lower epithelial layers; however, this analysis clearly highlighted the loss of EGFR expression in the upper suprabasal layers of E5KO rafts (Figure 4B; highlighted by an arrow).

EGFR levels were also determined by western blot of lysates from keratinocytes grown in high calcium media. In monolayer culture, NHK cells expressed low levels of EGFR, detectable only after longer exposure of the blot (Figure 4C; lanes 1-3). In contrast, EGFR expression was substantially increased in undifferentiated keratinocytes harbouring either WT or E5KO genomes compared to NHK (Figure 4C; compare lanes 1, 4 and 7). Upon differentiation, the level of EGFR protein declined moderately but was still detectable at 72 h in WT containing keratinocytes (Figure 4C; lanes 4-6). In the absence of E5, EGFR expression declined rapidly and was 90% lower than in WT cells at 72 h (Figure 4C; compare lanes 6 and 9 – p<0.0007). These data demonstrate that E5 maintains the increase in EGFR expression observed in suprabasal cells, but is not required for increased EGFR in the basal layer of the epithelium.

### Active EGFR is needed for cyclin B1 expression in differentiating keratinocytes

**Figure 5:**
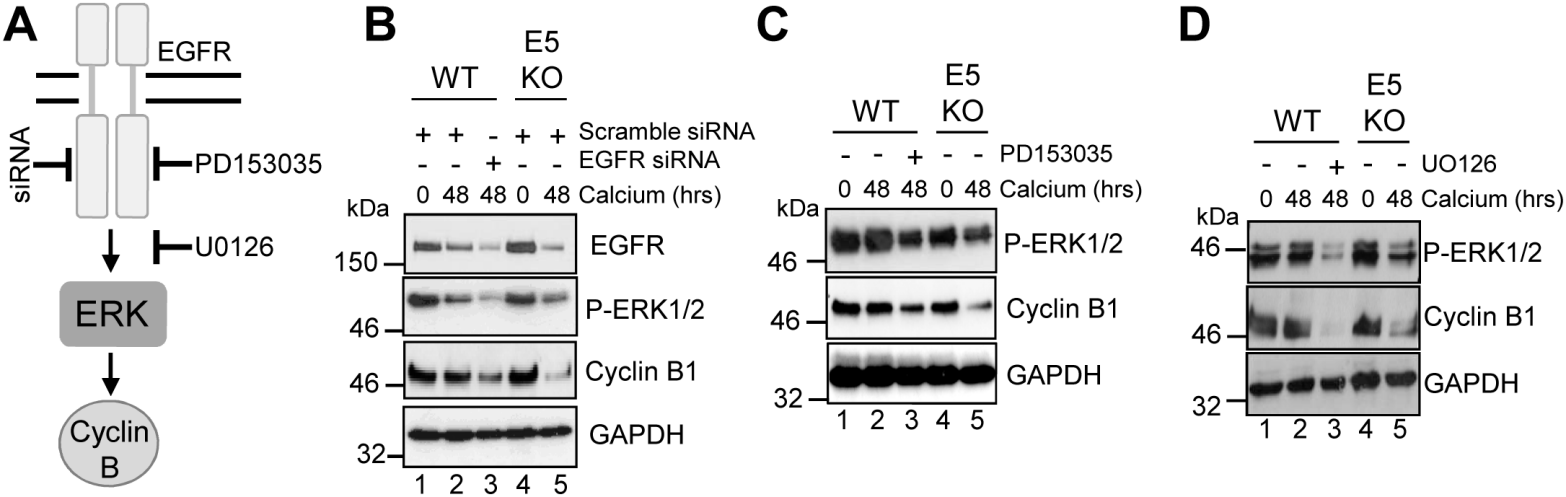
HPV18 requires EGFR activation to maintain cyclin B1 expression in differentiating cells **(A)** Schematic showing the EGFR/ERK signalling pathway and the targets of the siRNA and inhibitors used in this study. Mock treatedkeratinocytes were differentiated in high calcium media and lysed after 48 hours. In parallel, keratinocytes were treated with **(B)** scramble or EGFR specific siRNA, **(C)** an EGFR kinase inhibitor (2 μM PD153035), or **(D)** a Mek1/2 kinase inhibitor (20 μM UO126) during differentiation. All samples were analysed for cyclin B1 and phosphorylated ERK1/2 expression. GAPDH served as a loading control. Representative blots are shown from at least three independent biological repeats from two donor lines.

Next we studied the contribution of EGFR-ERK MAPK signalling to the cyclin B1 expression observed in differentiated HPV18 containing keratinocytes (Figure 5A). WT cells were transfected with a pool of commercially available, validated siRNA to deplete EGFR expression or a scrambled control, and cells cultured in high calcium media for 48 hours to induce differentiation. As expected, WT cells transfected with the scrambled control retained EGFR expression upon differentiation (Figure 5B; compare lanes 1 and 2). In contrast, transfection of the pool of EGFR specific siRNA resulted in a 50% reduction in EGFR levels in the differentiated cells (Figure 5B; compare lanes 2 and 3). The reduction in EGFR protein expression correlated with a 40% decrease in ERK1/2 phosphorylation, indicating that the signalling pathway was impaired. Importantly, a decrease in cyclin B1 expression to similar levels as in the E5KO cells was observed (Figure 5B; compare lanes 3 and 5). To investigate whether an active EGFR kinase was necessary for the increased cyclin B1 expression, keratinocytes harbouring WT genomes were grown in high calcium media for 48 hours in the presence of 2 μM of a specific and highly potent small molecule inhibitor targeting the intracellular EGFR kinase domain (PD153035 - [31]) (Figure 5C). Whereas the untreated WT cells retained cyclin B1 upon differentiation, cells treated with the EGFR inhibitor had reduced cyclin B1 expression similar to levels observed in the E5KO cells (Figure 5C; compare lanes 2 and 3 plus 3 and 5). The reduction in cyclin B1 expression correlated to reduced ERK1/2 phosphorylation, demonstrating that EGFR signalling was successfully inhibited (Figure 5C; compare lanes 2 and 3).

Similar results were observed with a specific small molecule inhibitor of Mek1/2 (UO126 – 20 μM - [32]), which inhibits activation of the ERK1/2 MAPK cascade (Figure 5D). These results indicate that activation of EGFR is necessary for the differentiation-dependent cyclin B1 expression in HPV18 genome containing cells. Notably, they also demonstrate that the blockade of this signalling pathway in cells harbouring the WT genomes lowers cyclin B1 levels to those observed in cells that lack E5 expression.

### HPV18 E5 reduces keratinocyte differentiation

**Figure 6:**
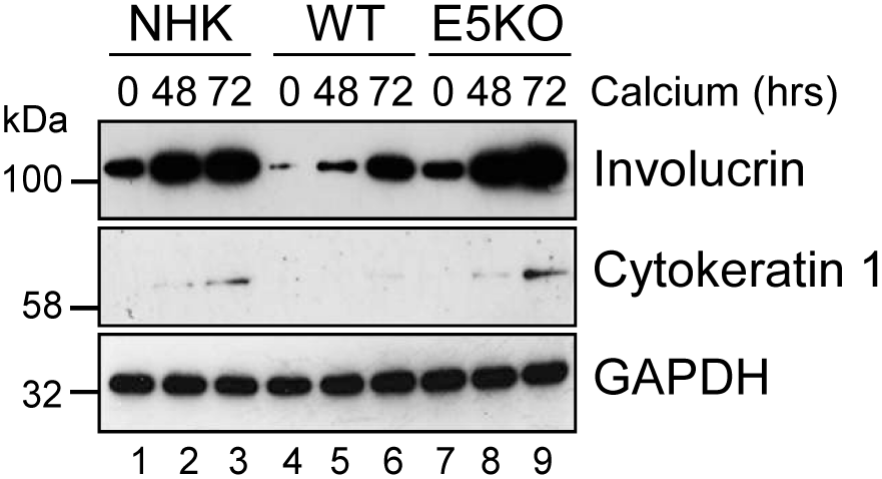
HPV18 E5 impairs keratinocyte differentiation Lysates of NHK, WT and E5KO keratinocytes subjected to high calcium differentiation were analysed for involucrin and cytokeratin 1 (K1) expression. GAPDH served as a loading control. Western blots are representative of three independent experiments in two donor lines.

Active EGFR is associated with a failure of differentiation in the epithelium. We hypothesized that enhanced EGFR expression might result in delayed keratinocyte differentiation. A defect in involucrin expression was observed in the E5KO cells by western blot of lysates from cells cultured in high calcium medium (Figure 6). In NHK and E5KO cells the levels of involucrin had risen markedly by 48 h growth in calcium, whereas in the WT cells levels of involucrin were 6-fold lower at 48h (p<0.02) and high levels of expression were achieved only after 72 h growth (Figure 6; compare lanes 2, 5 and 8). Similarly, levels of another differentiation marker – cytokeratin 1 – were perturbed in WT versus E5KO containing cells (Figure 6). These data indicate that E5 contributes to the HPV18 mediated suppression of epithelial differentiation.

### Negative regulation of the keratinocyte growth factor receptor (KGFR/FGFR2IIIb) by HPV18 E5

**Figure 7:**
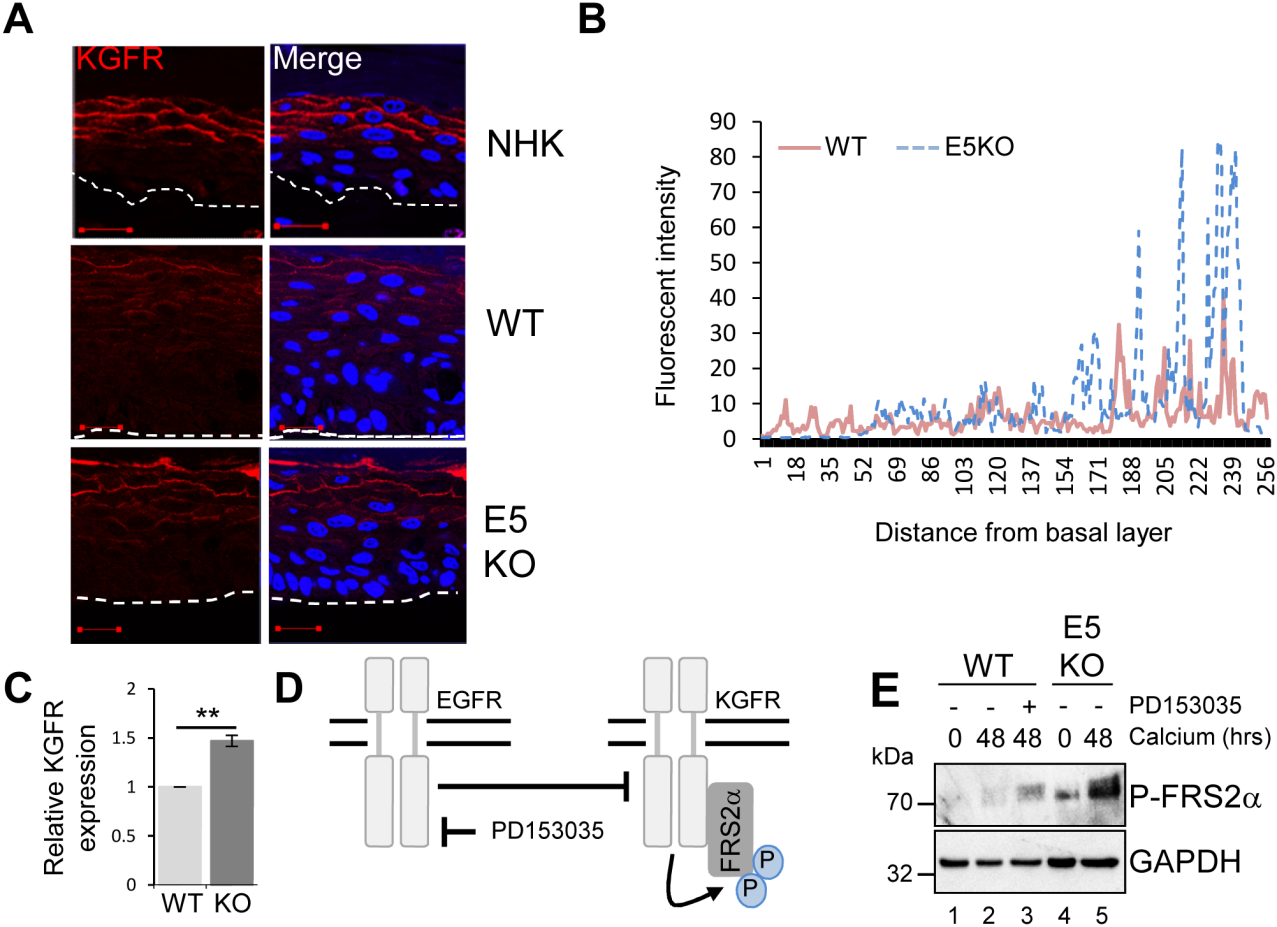
HPV18 E5 inhibits the KGFR signalling pathway **(A)** Organotypic raft cultures stained for KGFR expression (red) and nuclei stained with DAPI (blue). White dotted lines indicate the basal cell layer. Red scale bar represents 20 μm. Images are representative of staining from two donors. **(B)** Histogram analysis of the staining intensity from WT and E5KO rafts. Data was derived from 5 fields of view from three independent experiments, from two donors. **(C)** Graph showing relative KGFR mRNA expression in differentiated WT and E5KO keratinocytes after 96 hours incubation in high calcium media. Results were corrected for expression of an U6 loading control. The data is shown as a mean and standard deviation from three independent biological repeats from two donors. Significance as determined by Student’s t-test is shown as ^**^ = p<0.01. **(D)** Schematic showing the interplay between the EGFR/KGFR signalling pathway and the target of the inhibitor used in this study. **(E)** Mock-treated keratinocytes were differentiated in high calcium media and lysed after 48 hours. In parallel, keratinocytes were treated with an EGFR kinase inhibitor (2 μM PD153035), during differentiation. All samples were analysed for phosphorylated FRS2α expression. GAPDH served as a loading control. Representative blots are shown from at least three independent biological repeats from two donor lines.

To understand the mechanisms by which E5 regulates keratinocyte differentiation, we focused on the KGFR/FGFR2IIIb pathway. KGFR is a splice variant of the fibroblast growth factor receptor 2 (FGFR2IIIb) exclusively expressed in epithelial cells [33]. Reports have linked KGFR to skin homeostasis and as a regulator of the balance between proliferation and differentiation [9]. Mice lacking KGFR expression in skin epithelia display aberrant keratinocyte proliferation, impaired differentiation and are prone to the development of papilloma-like lesions [9]. First, levels of KGFR protein expression were examined by immunofluorescence staining in raft cultures (Figure 7A and 7B). As expected, KGFR expression was restricted to the suprabasal layers of the epithelium (Figure 7A and 7B). In NHK cells KGFR expression was only detected in the mid and upper suprabasal layers of the epithelium (Figure 7A). Overall levels of KGFR protein were reduced in WT containing cells, whilst the E5KO rafts showed a KGFR expression profile more similar to that observed in the NHK cells, with abundant KGFR seen in the mid and upper suprabasal layers (Figure 7A and 7B). HPV16 E5 has been suggested to impair KGFR transcription when overexpressed in HaCaT cells [34]. We were able to confirm that levels of KGFR mRNA were significantly lower in calcium differentiated WT compared to E5KO containing cells (Figure 7C).

### EGFR signalling suppresses KGFR expression

The EGFR pathway has been proposed to function as an inhibitor of KGFR expression [6, 35]. We tested whether KGFR expression could be repressed through EGFR signalling by treating cells harbouring WT genomes with the EGFR kinase inhibitor PD153035 over a 48 hour time course of differentiation with high calcium (Figure 7D). Cells were then lysed and assayed by western blot for KGFR activation. Due to inadequate antibody reagents, the phosphorylated form of FRS2α, a docking protein phosphorylated by the active form of KGFR, was used as a surrogate for active KGFR expression (Figure 7D) [33]. Levels of P-FRS2α were higher in differentiated E5KO compared to WT lysates (Figure 7E; compare lanes 2 and 5). Importantly, chemical inhibition of EGFR kinase activity (Figure 7D; compare lanes 2 and 3) resulted in an increase in the phosphorylated form of FRS2α, which correlates with active KGFR signalling.

### KGFR signalling is impaired in cells harbouring HPV18 genomes

**Figure 8:**
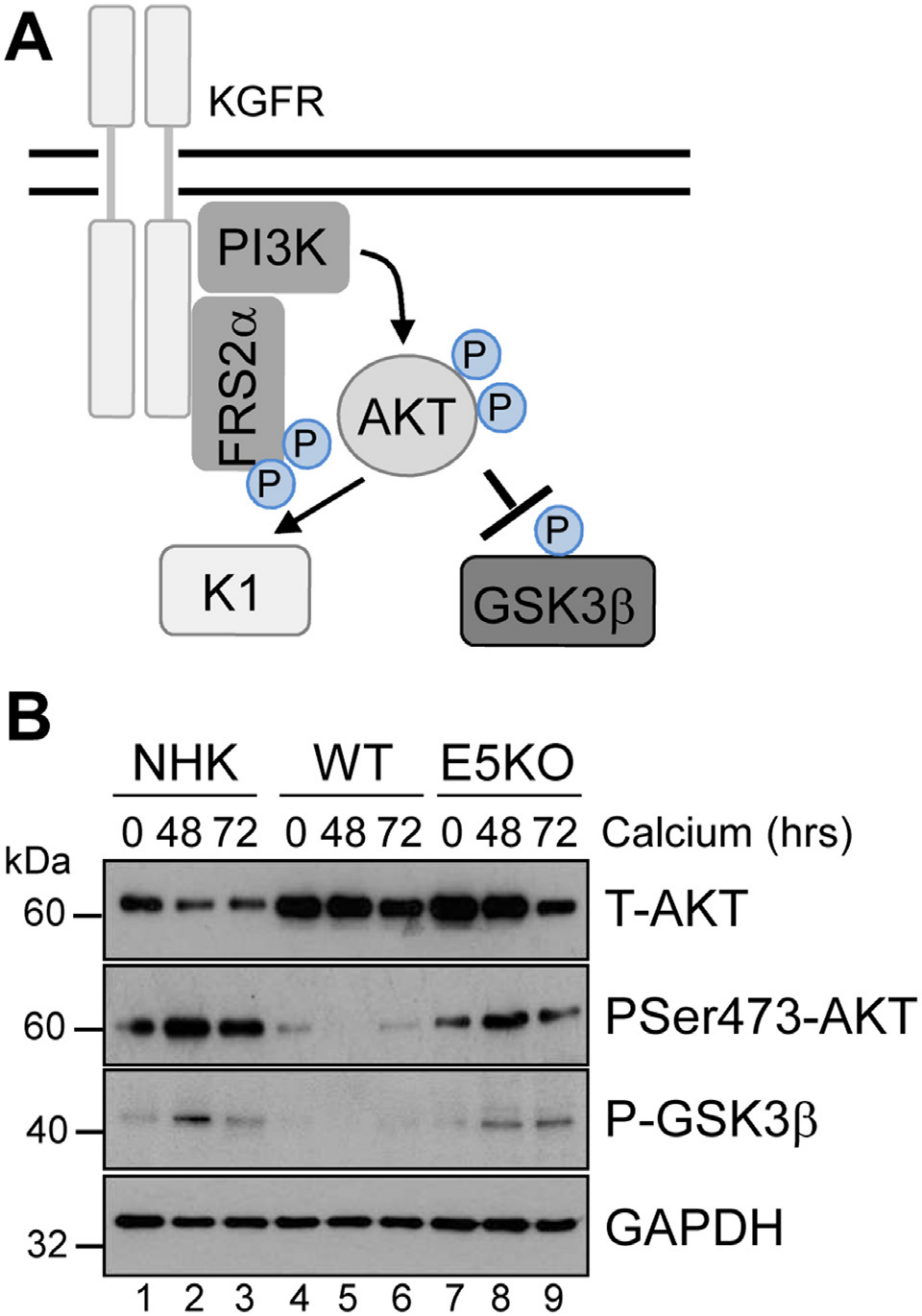
HPV18 E5 inhibits downstream targets of the KGFR signalling pathway **(A)** Schematic showing the KGFR downstream signalling pathway including the KGFR substrate FRS2a and downstream kinases AKT and GSK3β. **(B)** Lysates of NHK, WT and E5KO keratinocytes subjected to high calcium differentiation were analysed for p-AKT, total AKT and p-GSK3β. GAPDH served as a loading control. Western blots are representative of three independent experiments in two donor lines

AKT is a master serine/threonine kinase, which acts as a major downstream effector of KGFR during keratinocyte differentiation. Upon KGFR mediated activation of phosphatidylinositol 3-kinase (PI3-kinase), and the subsequent generation of phosphatidylinositol 3,4,5-trisphosphate, AKT is recruited to the plasma membrane, where it is phosphorylated and activated [36]. AKT phosphorylates a number of substrates in the cytoplasm and nucleus associated with keratinocyte differentiation and survival e.g. GSK3β (Figure 8A) [37, 38]. To understand the implications of the loss of KGFR expression on down-stream signalling pathways, we examined the expression and post-translational modification of AKT in our HPV genome-containing cell lines upon differentiation in high calcium medium. The levels of total AKT diminished slightly upon differentiation, although they remained higher in HPV18 containing cells compared to NHK controls and the loss of E5 expression did not impact on the increase in AKT (Figure 8B; compare lanes 1, 3 and 7). Whilst the level of total AKT protein decreased upon differentiation, there was a differentiation-dependent increase in Ser473 AKT phosphorylation in NHK cells (Figure 8B; compare lanes 1 with 2 and 3). In contrast, the level of phosphorylated Ser473 AKT was substantially reduced in the WT containing cells, both in undifferentiated and in differentiated cells (Figure 8B; compare lanes 1 and 4, 3 and 6). The loss of E5 protein reinstated the temporal pattern of differentiation-dependent AKT phosphorylation (14 fold increase in P-AKT in the E5KO compared to WT at 72h – p<0.02), although the level of phosphorylation was lower than in the NHK cells (Figure 8B: compare lanes 1, 4 and 7 and 3, 6 and 9).

A similar loss in the differentiation-dependent increase in GSK3β phosphorylation was observed in WT genome-containing cells (Figure 8B; compare lanes 1-3 and 4-6). Again, E5KO cells recapitulated the pattern of GSK3β phosphorylation observed in NHK cells but to lower levels (Figure 8B; compare lanes 1-3 with 7-9). Together these data indicate that HPV18 E5 reduces differentiation-dependent AKT activation in keratinocytes.

### Pharmacological blockade of KGFR compensates for the loss of E5 and delays keratinocyte differentiation

**Figure 9:**
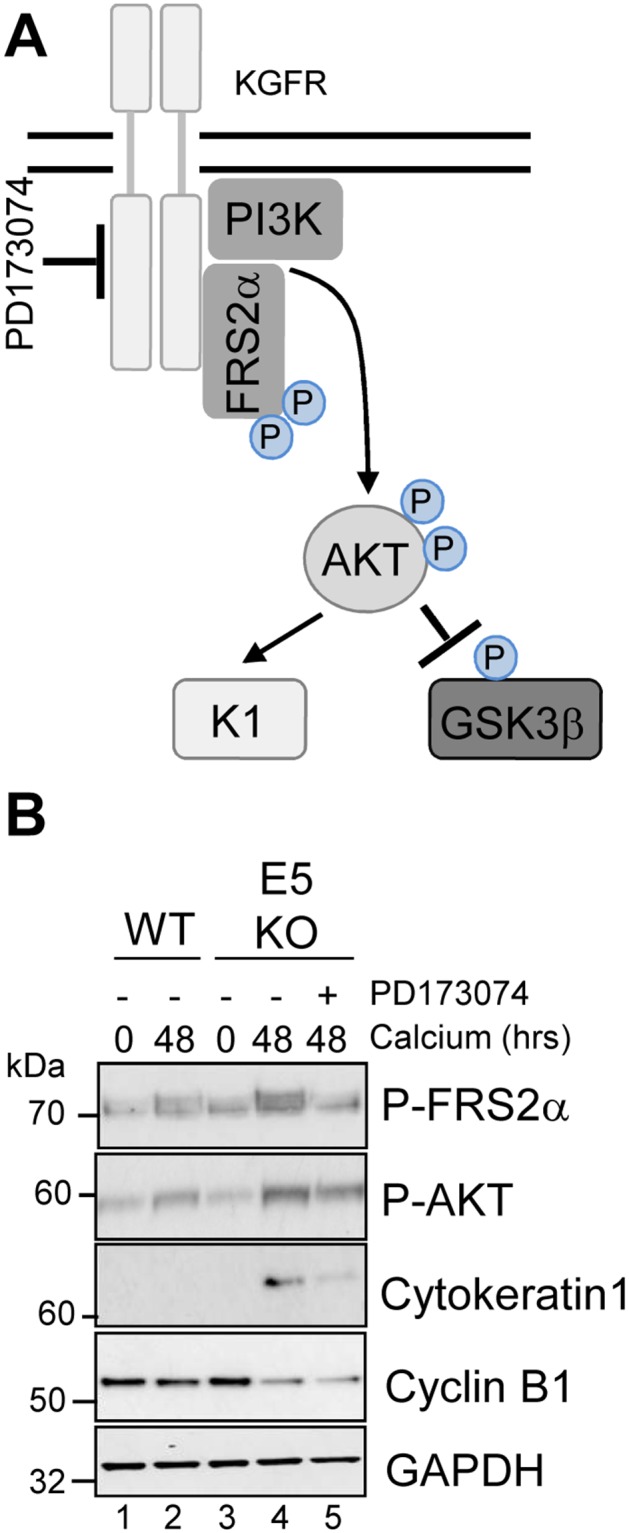
Pharmacological inhibition of KGFR activity compensates for loss of E5 and impairs keratinocyte differentiation **(A)** Schematic showing the KGFR signalling pathway and target of the pharmacological inhibitor. **(B)** Control (vehicle only) keratinocytes were differentiated in high calcium media and lysed after 48 hours. Parallel cultures of E5KO keratinocytes were treated with 30 nM of a KGFR kinase inhibitor (PD173074). Lysates were analysed for the phosphorylated forms of FRS2α, AKT, GSK3α/β, cytokeratin 1 (K1) and cyclin B1 by immunoblot.

To investigate whether the failure of E5KO cells to impair differentiation was due to an inability to down-regulate KGFR signalling, we determined whether pharmacological inhibition of the KGFR pathway might compensate for loss of E5. To inhibit KGFR kinase activity, E5KO cells were differentiated in the presence of PD173074 (30 nM), a potent small molecule inhibitor targeting FGFR2 kinases (Figure 9A) [39]. We first confirmed the effects of PD173074 by assessing the phosphorylation status of the adaptor protein FRS2α. As expected, levels of FRS2α phosphorylation were greater in the E5KO cells upon differentiation compared to WT (Figure 9B, compare lanes 2 and 4), indicating increased KGFR activity. The addition of PD173074 reduced the levels of FRS2α phosphorylation in the E5KO cells, and this effect correlated with a decrease in AKT phosphorylation and reduced expression of cytokeratin 1 (Figure 9B).

We were intrigued to test whether pharmacological blockade of KGFR might also increase cyclin B1 expression in the E5KO cells. Treatment with the KGFR inhibitor had no impact on cyclin B1 expression in the E5KO cells (Figure 9B). This indicates that the E5 mediated increase in cyclin B1 expression is either upstream of KGFR repression or within a separate signalling network. We conclude that blockade of KGFR in cells harbouring HPV18 E5KO genomes rescues the differentiation phenotype associated with the absence of E5, but cannot prevent the reduction of cyclin B1 expression.

### AKT is essential for the E5-mediated reduction in keratinocyte differentiation

**Figure 10:**
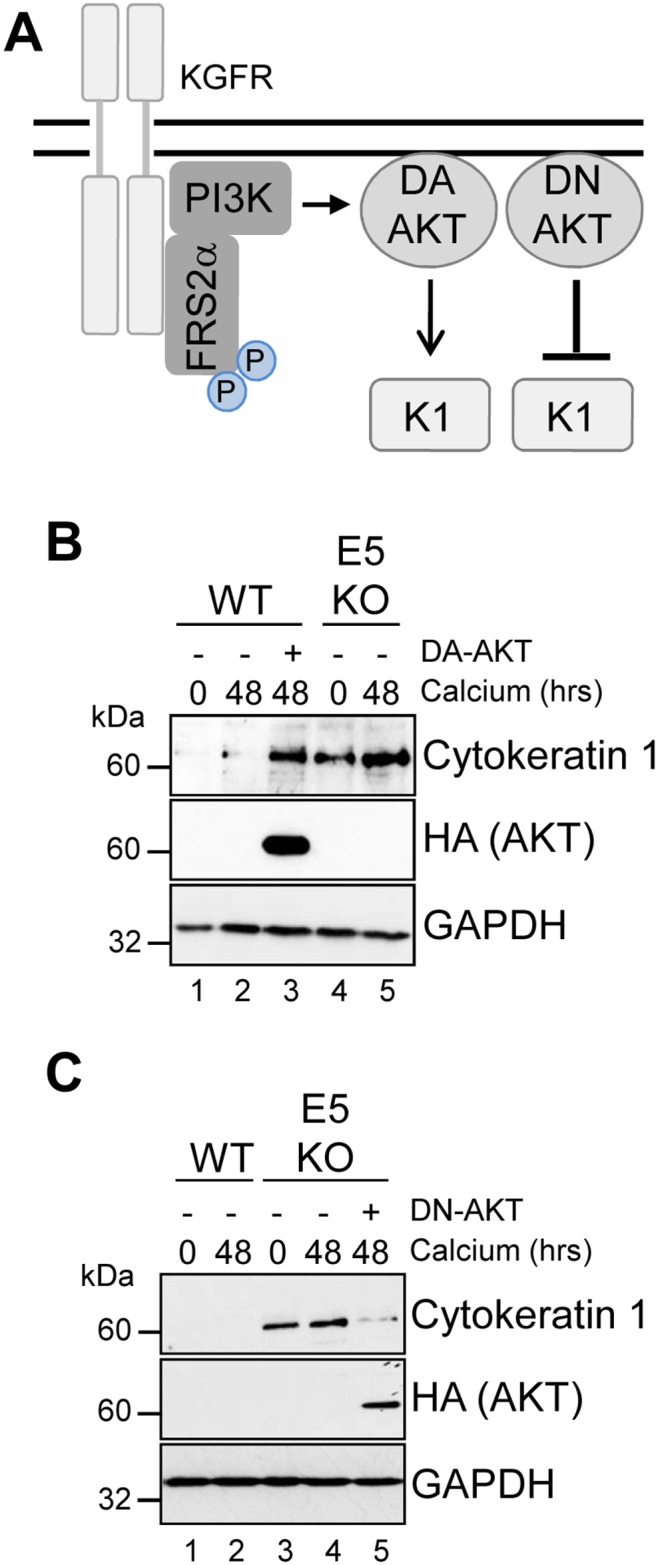
AKT is essential for the modulation of keratinocyte differentiation by HPV18 **(A)** Schematic showing the KGFR signalling pathway and the effects of the dominant active (DA) and negative (DN) AKT. HPV18 genome containing keratinocytes were infected with empty retrovirus, or with retroviruses encoding DA AKT **(B)** or DN AKT **(C)**. Cells were differentiated in high calcium media for 48 hours prior to lysis and analysed for expression of cytokeratin 1 (K1) to assess differentiation. Expression of the exogenous AKT was confirmed using an antibody against the HA epitope and GAPDH served as a loading control.

Our data indicates that suppression of KGFR by E5 was necessary to reduce keratinocyte differentiation, and raised the possibility that the regulation of downstream components of KGFR signalling might be critical for keratinocyte differentiation and the productive virus life cycle. One such effector is AKT, which plays a functional role in the construction of the suprabasal layers [38]. We sought to determine the contribution of AKT inhibition by E5 to keratinocyte differentiation (Figure 10A). We asked whether a myristoylated form of AKT1 – the predominant isoform of AKT in human keratinocytes [38] – that is targeted to the membrane independently of PI3-kinase activity could overcome the delay in early differentiation exhibited in keratinocytes harbouring HPV18 genomes. WT genome containing cells were transduced with a retrovirus encoding AKT1 containing an amino terminal HA epitope tag engineered to contain the myristoylation signal sequence from the c-Src tyrosine kinase [40], and subsequently cultured in high calcium containing media for 48 hours. Analysis of the protein lysates by western blot confirmed the low levels of the differentiation marker cytokeratin 1 in WT cells compared to those lacking E5 (Figure 10B; compare lanes 2 and 5). In contrast, cells expressing constitutively active AKT1 overcame the HPV18 mediated block and expressed levels of cytokeratin 1 similar to the E5KO cell line (Figure 10B; compare lanes 2 and 3). Western blotting with an antibody recognizing the HA epitope confirmed expression of the exogenous AKT1 in the appropriate sample.

If AKT is a critical host target for E5 function, then we anticipated that the introduction of a dominant negative form of this protein might compensate for the lack of E5 and delay keratinocyte differentiation (Figure 10A). To test this, E5KO cells were transduced with a retrovirus encoding a kinase inactive version of the myristoylated AKT1 protein and differentiated for 48 hours in the presence of high calcium. Western blotting with an anti-HA antibody confirmed exogenous AKT1 expression (Figure 10C). As predicted, the differentiation-induced increase in cytokeratin 1 expression associated with the E5KO cells was significantly reduced in cells expressing the dominant negative AKT1, almost to levels observed in cells harbouring the WT genomes (Figure 10C; compare lanes 4 and 5). Thus, E5 function to delay keratinocyte differentiation correlates with a deregulation of AKT activity.

## DISCUSSION

This study provides a comprehensive analysis of E5 function during productive infection by HPV18; the second most prevalent high-risk type. We found little impact of the loss of E5 on the HPV life cycle in undifferentiated cells, in agreement with previous studies in keratinocyte-based model systems of the HPV16 and HPV31 viruses [21, 22]. E5KO genomes efficiently established episomes and the genome-containing cells showed no growth disadvantage under these undifferentiated conditions. One possible explanation for these findings might lie in the polycistronic nature of HPV gene expression. Whilst recent transcript mapping studies show that E5 is present on abundant transcripts, it is often last in the polycistronic message and as such it may be inefficiently expressed in undifferentiated conditions [28]. Unfortunately, the absence of an effective E5 antibody makes this difficult to test. Alternatively, it is possible that in basal cells an additional early protein (e.g. E6 or E7) compensates for the loss of E5.

This study clearly demonstrates that E5 is required to effectively drive cell cycle re-entry in suprabasal cells, as judged by repression of both on-going host cell DNA synthesis and elevated cytoplasmic cyclin B1 expression in the suprabasal regions of E5KO rafts. Given that viral DNA amplification is thought to be initiated in cells with cytoplasmic cyclin B1 [29], we studied whether E5 might contribute to this process and observed no reduction in the number of cells supporting virus genome amplification upon differentiation. In fact, there was no significant impact of E5 loss on HPV18 late gene expression, as tested by immunostaining of raft cultures for E4 and L1 proteins. Thus, our data fit the recently described model, [23], whereby high levels of viral genome amplification are necessary for late protein expression. Our findings are in agreement with the HPV16 E5 knockout model, which also saw no decrease in late protein expression [21], but are in disagreement with Fehrmann and colleagues who observed a significant decrease in E4 expression in HPV31 E5KO cells [22]. Whether these differences are due to alternative experimental techniques or are a genuine type-specific difference between E5 functions awaits further study. With regards to the latter, HPV18 is predominantly associated with adenocarcinomas of the cervix, whereas HPV16 and HPV31 have a strong association with squamous carcinomas; these differences in pathogenesis may indicate differences in life cycle biology. Indeed, it has recently been shown that E4 function is markedly different between HPV16 and HPV18 [41]. Thus, our data suggest that E5 is required to modulate the host cell milieu to generate cell cycle progression at particular stages of the productive HPV18 life cycle, but perhaps its loss can be compensated for by other viral protein functions, including E7, E6 or E4.

Whilst the requirement for E5 in cell cycle progression was observed in the previous studies [21, 22], neither study was able to provide a definitive link between cell cycle progression and EGFR signalling. This was surprising given the body of literature demonstrating a link between E5 expression and EGFR activation coupled with the understanding that EGFR-ERK MAPK signalling is a major driver of cyclin B1 expression in keratinocytes [5, 6]. We observed enhanced EGFR expression and activity in differentiated keratinocytes containing HPV18 genomes compared to parental control cells. Absence of E5 correlated with reduced suprabasal EGFR expression but had no impact on EGFR expressed in the basal compartment. These data are consistent with the notion that E5 is not expressed and/or required in basal cells and that in these cells another HPV encoded protein increases EGFR expression. Through the use of siRNA depletion we were able to demonstrate that the increased EGFR expression is necessary for cyclin B1 expression in differentiating keratinocytes. Moreover, small molecule inhibitors inactivating the EGFR kinase domain demonstrate that an active EGFR is required for this process. A prevailing model for E5-mediated EGFR expression suggests a mechanism linked with endosome deacidification or modulation of intracellular endosomal trafficking, whereby activated EGFR are re-routed from a degradative pathway into recycling endosomes, from which they are trafficked back to the plasma membrane. This might be accomplished by impaired endosome acidification resulting from either interaction with the vacuolar ATPase [42], modulation of endosome maturation [43] or involve the recently described viroporin activity of E5 [13].

Proliferative signalling is also known to suppress expression of proteins that positively regulate epithelial differentiation. We discerned a significant defect in differentiation marker expression in cells harbouring the HPV18 genomes. Despite the increase in proliferative potential in cells harbouring HPV16 and HPV31 genomes, no apparent differences were observed in differentiation marker expression in the absence of E5 expression [21, 22]. These findings do not correspond with the disturbances in epithelial differentiation observed in the HPV16 E5 transgenic mouse model [19]. It is unclear why the HPV18 life cycle model shows such an overt differentiation phenotype in comparison to comparable studies of HPV16 and 31. One explanation might lie in the level of proliferative signalling controlled by E5 in HPV18 containing cells. Since EGFR signalling suppresses differentiation it is possible that HPV18 E5 is more effective at suppressing these pathways than E5 from other high-risk types studied to date.

Since the KGFR pathway is a known regulator of the early stages in suprabasal differentiation, we tested whether manipulation of KGFR might contribute to the defect in differentiation observed in primary keratinocytes harbouring the HPV18 genome. In support of this, the KGFR pathway is inhibited in HaCaT cells constitutively expressing isolated HPV16 E5 [44]. KGFR signalling was impaired in cells harbouring the WT HPV18 genomes. In particular, we studied the KGFR effector AKT, a serine/threonine kinase that regulates a number of events in suprabasal cells. Levels of AKT phosphorylation and kinase activity were suppressed in cells harbouring HPV18. We were able to demonstrate the importance of the KGFR-AKT pathway for differentiation using a combination of small molecule inhibitors and retrovirus encoded dominant active and negative AKT proteins. Notably, treatment of E5KO cells with a KGFR kinase inhibitor or over-expression of a dominant negative AKT protein compensated for the loss of E5 during differentiation and exhibited a similar WT HPV18 differentiation marker expression profile. In addition, overexpression of a dominant active AKT protein in cells harbouring WT viral genomes prevented the virus-associated delay in differentiation. Importantly, E5KO cells retain WT levels of E6 and E7 expression, which have been previously shown to potently suppress keratinocyte differentiation [24, 26, 46–51]. These data imply that AKT is a critical regulatory protein in the keratinocyte differentiation program. It will be of great interest to identify the targets of AKT that function to regulate differentiation in the context of HPV infection. One candidate is GSK3β, the phosphorylation of which is decreased in cells harbouring WT genomes. Interestingly, it has recently been shown that GSK3 β was more active in cells containing HPV31 [45]. Since phosphorylation of GSK3 β by AKT serves to inhibit the function of this protein our data corroborates these findings and implies that targeting of the AKT-GSK3β axis is a common feature between the high-risk viruses HPV18 and HPV31.

A number of HPV early protein knockout models have been used to great effect to dissect the apparent contribution made by each protein to the productive life cycle. A major observation from these studies is the level of redundancy of function, in particular between the three oncogenes. For example, loss of E7 impacts upon S-phase progression in suprabasal cells and loss of both E6 and E7 expression correlates with a dysfunctional delay to keratinocyte differentiation. We demonstrate that E5 also targets these processes. We do not believe that our observations are due to any disturbance in E6 and/or E7 expression in our E5 knockout model for three reasons. Firstly, no significant difference was observed in E6 and E7 expression between the WT and E5KO cells. Secondly, several of our findings have been noted using isolated E5 expression, including S-phase progression [52], EGFR hyperactivation [13] and suppression of KGFR transcription [34, 53]. Finally, the E5KO phenotype is subtler than either the E6 or E7 knockout phenotypes. For example, we observe no impact on genome maintenance, seen in the absence of E6 and despite a significant reduction in suprabasal DNA synthesis we see no effect on viral DNA amplification or late protein expression, both of which are abrogated in the absence of E7. Given the temporal nature of HPV protein expression it is possible that specific early proteins target similar pathways at distinct stages during epidermal differentiation. This would explain why little impact is seen in the basal cells of the E5KO keratinocyte based models. It is also possible that early proteins fulfil distinct roles in each papillomavirus type, and that each HPV type might modulate similar pathways using distinct early proteins targeting divergent host proteins. Large-scale proteomic screens of early protein binding partners from a range of HPV types may aid in addressing these possibilities [54].

**Figure 11:**
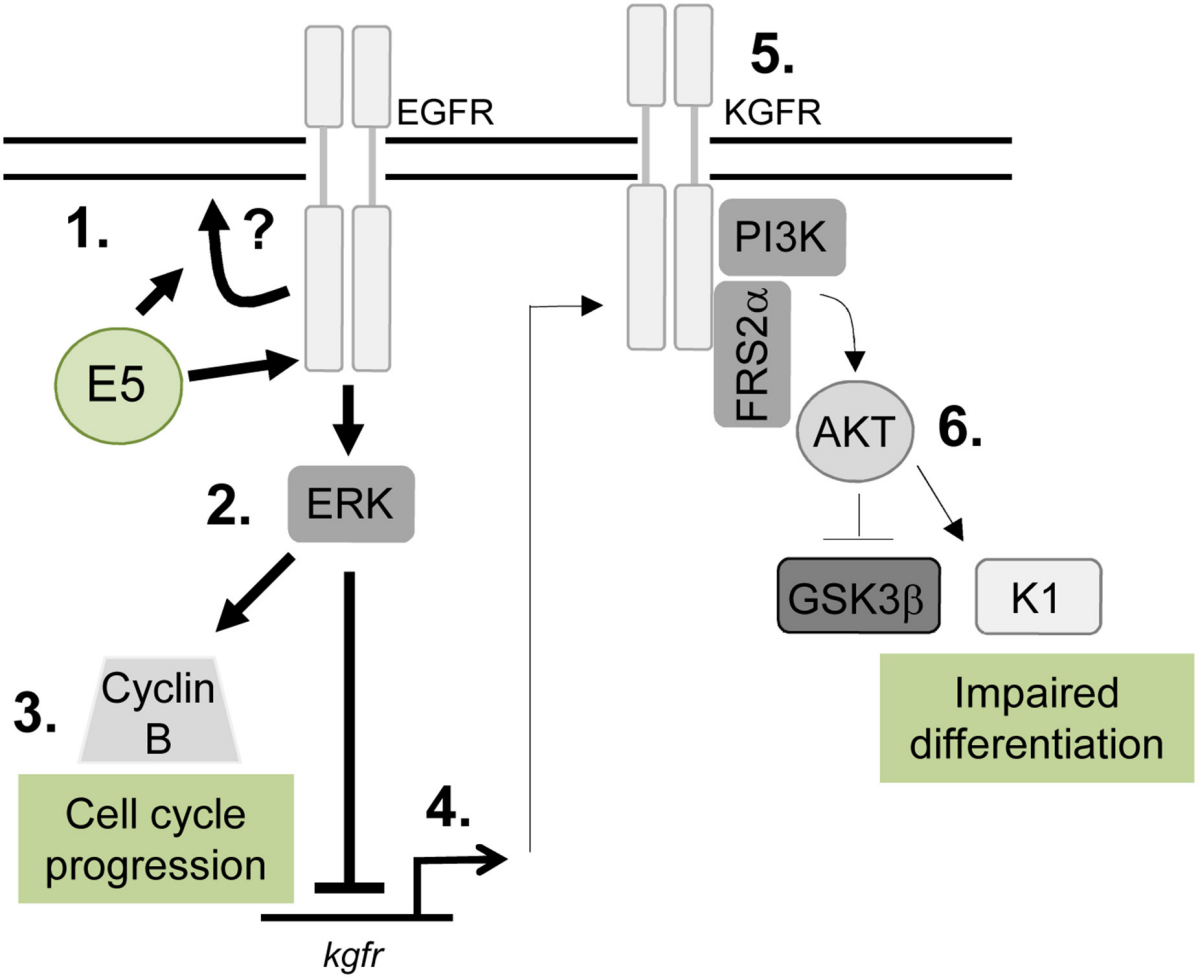
Schematic model depicting the proposed role of EGFR signalling in the E5-mediated manipulation of proliferation and differentiation pathways during the virus life cycle E5 expression results in enhanced EGFR surface expression and activation, through a process that might require endosome recycling (1). This increases ERK/MAPK activity (2), resulting in activation of substrates, which include cell cycle associated proteins e.g. cyclin B (3). EGFR can suppress keratinocyte differentiation, by inhibiting a number of targets including the KGFR pathway. KGFR transcription is suppressed in E5 expressing cells (4) and KGFR signalling is markedly reduced (5). As a consequence, targets of KGFR including Akt are suppressed, culminating in loss of expression of a number of spinous associated differentiation markers e.g. cytokeratin 1 (K1) (6). As a result keratinocyte differentiation is impaired in cells expressing E5.

This study demonstrates an unanticipated role for E5 in the HPV18 life cycle and identifies key epithelial pathways manipulated by this enigmatic oncoprotein. We show a unifying role for EGFR signalling in E5 function both as an enhancer of cell cycle progression and a repressor of epithelial differentiation (Figure 11). Given the importance of EGFR activation for E5 function it will be essential in the future to determine how E5 activates the EGFR and whether the importance of EGFR signalling is a shared feature of other HPV E5 proteins. Future studies should also focus on understanding the molecular interplay between the EGFR and KGFR pathways in HPV containing keratinocytes. Finally, it has been reported that E5 contributes to the immune evasion strategy of HPV by interfering with host MHC proteins in keratinocytes. Our model system provides an ideal opportunity to study this additional role during the productive HPV18 life cycle.

## MATERIALS AND METHODS

### Small molecule inhibitors

The quinazolone PD153035 is a specific and highly potent inhibitor of the EGFR kinase domain [31]. It was added to cells at a final concentration of 1 μM. UO126 is a selective Mek1/2 inhibitor, and is used to inhibit activation of ERK1/2 [32]. It was added to cells at a final concentration of 20 μM. PD173074 was used to inhibit the kinase domain of the FGFR kinase member KGFR [39] and added to cells at a final concentration of 30 nM. PD153035, UO126 and PD173074 were purchased from Calbiochem.

### Generation of E5 knockout-HPV18 pGEMII

pGEMII plasmid containing the complete HPV18 genome (accession number: NC 001357) was a gift from F. Stubenrauch (University of Tübingen, Germany). A translation termination codon (TTA>TAA) was engineered immediately after the E5 start codon by site directed mutagenesis to include a translation termination codon (TTA>TAA) immediately after the E5 start codon using mutagenic primers FwdE5stopmutant and RevE5stopmutant (Supplementary Table 1). Briefly, 10 ng of DNA was cycled 20 times with 240 ng of each mutagenic primer in a 50 μl reaction containing 1x KOD PCR buffer, 0.3 mM of each dNTP and 1 U *Thermococcus kodakaraenis* KOD polymerase (Roche). Stbl2 cells (Invitrogen) were transformed with *Dpn*I-digested PCR product and used to amplify the plasmid DNA. Sequencing using GATC Biotech Ltd services was used to sequence the entire HPV18 genome to confirm mutagenesis of E5 and verify that no additional nucleotide changes had been generated during the mutagenesis protocol (primer sequences can be found in Supplementary Table 1).

### Cell transfection

The transfection of primary human foreskin keratinocytes (NHK) isolated from neonate foreskin tissues (ethical approval no. 06/Q1702/45 for the collection and use of neonate foreskin tissue) was performed in S. Roberts’ laboratory as described previously [26, 55]. To account for donor-specific effects, cell lines harbouring the HPV18 genomes were generated in NHK isolated from two donors. Briefly, plasmids containing the wild type (WT) and E5 knock-out (E5KO) were digested with *Eco*RI to release the genome, which was then re-circularised with T4 DNA ligase. The genomes were co-transfected with a plasmid encoding resistance to neomycin (pcDNA3.1, Invitrogen) into low passage NHK in serum free medium (Invitrogen). The cells were harvested 1 day later and seeded onto a layer of γ-irradiated J2-3T3 fibroblasts and selected with G418 antibiotic in complete E medium containing 5% foetal calf serum (FCS, Lonza) and 5 ng/mL epidermal growth factor (BD BioSciences) for 8 days. Cell colonies that emerged were pooled and expanded on γ-irradiated J2-3T3 fibroblasts in complete E medium.

### siRNA transfection

A pool of 4 siRNA against EGFR (Targeting different regions of the mRNA) (Qiagen catalogue number GS1956) were transfected into HPV18 containing primary keratinocytes using lipofectamine 2000 transfection reagent (Life technologies) and incubated for 24 hours. The cells were then grown in serum free keratinocyte media containing 1.8mM CaCl_2_ for a further 48 hours.

### Organotypic raft cultures

Keratinocytes containing HPV18 WT and E5KO genomes were grown in organotypic raft cultures by seeding the keratinocytes onto collagen beds containing J2-3T3 fibroblasts. Once confluent the collagen beds were transferred onto metal grids and fed from below with FCS-containing E media lacking EGF. The cells were allowed to stratify for 13 days before fixing with 4% formaldehyde in E media. The rafts were paraffin-embedded and 4 μm tissue sections prepared (Propath UK, Ltd., Hereford, UK). Cellular DNA synthesis was analysed by the addition of 20 μM BrdU to the medium 16-18 hours prior to fixation. To detect BrdU-labelled nuclei, sections were immunostained with a fluorescein isothiocynate-conjugated antibody specific for BrdU (Invitrogen). Sections were stained with haematoxylin and eosin to assess morphology. For analysis of involucrin (SY5, Santa Cruz Biotechnology (sc-21748)), cyclin B1 (H-433, Santa Cruz Biotechnology (sc-752)), HPV18 E4 (R424, [27]), HPV18 L1 (5A3, Novacastra Laboratories), EGFR (R-1, Santa Cruz Biotechnology) and KGFR (H-80, Santa Cruz Biotechnology) protein expression, the formaldehyde-fixed raft sections were treated with the sodium citrate method of antigen retrieval. Briefly, sections were boiled in 10 mM sodium citrate with 0.05% Tween-20 for 10 minutes. Sections were incubated with appropriate antibodies and immune complexes visualized by using Alexa 488 and 594 conjugated secondary antibodies (Invitrogen). The nuclei were counterstained with the DNA stain 4’,6-diamidino-2-phenylindole (DAPI) and mounted in Prolong Gold (Invitrogen).

To detect nuclei positive for viral DNA amplification, organotypic raft culture sections were probed with a biotin-conjugated HPV DNA probe specific for the high-risk HPV types [26] using Leica BOND-Max technology as described by the manufacturer (Leica Microsystems, Milton Keynes) and imaged with a light microscope (performed by Human Biomaterials Resource Centre (University of Birmingham).

### Southern blot analysis

Total genomic DNA was extracted from cell culture by phenol-chloroform extraction. DNA (5 μg) was treated with *Dpn*1 to digest residual input DNA and either *Bgl*II or *Eco*RI to analyse the physical status of the HPV18 genomes. There are no *Bgl*II sites in the HPV18 genome. The digested DNA was analysed on a 0.8% agarose gel and DNA transferred to GeneScreen™ nylon membrane. Complete HPV18 genome was released from the pGEMII backbone vector by *Eco*RI digestion, purified and labelled with [α-^32^P]-CTP. The membrane was incubated with this radiolabelled linear probe at 42°C overnight. Following washing the membrane was exposed to auto-radiograph film.

### High calcium differentiation assay

Untransfected NHK and HPV18 containing keratinocytes were grown in complete E media until 90% confluent. Media was changed to serum free keratinocyte media without supplements (SFM medium, Invitrogen) containing 1.8 mM calcium chloride. Cells were maintained in this media for between 48 - 72 hours before lysis and analysis.

### Western blotting

Total protein was extracted from keratinocytes in Leeds lysis buffer [56] and resolved by SDS-PAGE (10-15% Tris-Glycine), transferred onto Hybond nitrocellulose membrane (Amersham biosciences) and probed with antibodies specific for phosphorylated FRS2α (3861, Cell Signaling Technology), cyclin B1 (H-433, Santa Cruz Biotechnology), involucrin (SY5, Santa Cruz Biotechnology), HPV18 E6 (G-7, Santa Cruz Biotechnology), HPV18 E7 (8E2, Abcam (ab100953), AKT (9272, Cell Signaling Technology), phospho-AKT Ser473 (D9E, Cell Signaling Technology), HA (HA-7, Sigma H9658), cytokeratin 1 (Poly19056, Covance), phospho-ERK1/2 (43705, Cell Signalling Technology), GAPDH (G-9, Santa Cruz Biotechnology), phospho-GSK3α/β (9336, Cell Signalling Technology) and EGFR (R-1, Santa Cruz Biotechnology). Immunoblots were visualized with species-specific HRP conjugated secondary antibodies (Sigma) and ECL (Thermo/Pierce).

### Retrovirus transduction

pLNCX AKT vectors (Addgene 9005, 9006 [40]) were transfected into HEK 293T cells with murine retrovirus envelope and GAG/polymerase plasmids (kindly provided by Professor Greg Towers, University College London) using PEI transfection reagent. After 48 hours the media was removed from the HEK 293T cells and added to keratinocytes for 3 hours. After this time, the complete E media was replaced and the cells incubated for 16 hours.

### Quantitative real-time PCR

Total RNA was extracted from NHK using the E.Z.N.A. Total RNA Kit I (Omega Bio-Tek) according to the manufacture’s protocol. Total RNA (1 μg) was reverse transcribed with a mixture of random primers and oligo(dT) primers using the qScript™ cDNA SuperMix (Quanta Biosciences) according to instructions. Q-RT-PCR was performed using the QuantiFast SYBR Green PCR kit (Qiagen) and primers specific to KGFR (Supplementary Table 1). The PCR reaction was conducted on a Corbett Rotor-Gene 6000 (Qiagen) as follows: initial activation step for 10 min at 95°C and a three-step cycle of denaturation (10 sec at 95°C), annealing (15 sec at 60°C) and extension (20 sec at 72°C) which was repeated 40 times and concluded by melting curve analysis. The data obtained was analysed according to the ΔΔ C_t_ method [57] using the Rotor-Gene 6000 software. U6 served as normaliser gene.

### Analysis of raft staining

Analysis of staining intensities was performed using the image J software. Cross sections of the raft sections were plotted using the histogram function on the software. Histograms represent cross sections from 5 fields of view from three independent experiments.

## SUPPLEMENTARY MATERIALS TABLE


