# Supplementary material for: Human papillomavirus type 18 E5 oncogene supports cell cycle progression and impairs epithelial differentiation by modulating growth factor receptor signalling during the virus life cycle
Source: Oncotarget. 2017 Oct 6;8(61):103581–600. doi: 10.18632/oncotarget.21658 (PMC5732752; doi:10.18632/oncotarget.21658)
Supplement: Supplementary file 1 [file oncotarget-08-103581-s001.pdf]

Human papillomavirus type 18 E5 oncogene supports cell cycle progression and impairs epithelial differentiation by modulating growth factor receptor signalling during the virus life cycle

SUPPLEMENTARY MATERIALS

Supplementary Table 1: List of all primer sequences used in this study

Name	Sequence 5’ – 3’	Application
FwdE5stopmutant	gctgtagtaccaatatgtAatcacttatt	site directed mutagenesis
RevE5stopmutant	acatattggtactacagcatatgtattac	site directed mutagenesis
HPV18 seq A	atagacagccaaatacag	HPV18 genome sequencing
HPV18 seq B	actgtgcggcgtgtagca	HPV18 genome sequencing
HPV18 seq C	actatatcttctgcctct	HPV18 genome sequencing
HPV18 seq D	aggtgttggccttagtgg	HPV18 genome sequencing
HPV18 seq E	aggtgttggccttagtgg	HPV18 genome sequencing
HPV18 seq F	attgcattgtatggtatg	HPV18 genome sequencing
HPV18 seq G	accgaaaacggtcgggac	HPV18 genome sequencing
HPV18 seq H	atggagttaatcatcaac	HPV18 genome sequencing
HPV18 seq I	atagtggctatggctgtt	HPV18 genome sequencing
HPV18 seq J	agtagtgttgcagcacta	HPV18 genome sequencing
KGFRfwd	CAGGGGTCTCCGAGTATGAA	qRT-PCR
KGFR rev	TCTAAAGGCAACCTCCGAGA	qRT-PCR
U6 fwd	CTC GCT TCG GCA GCA CA	qRT-PCR
U6 rev	AAC GCT TCA CGC ATT TGC	qRT-PCR
